# Pulsed electric field ablation process—a study of the effect of different shapes (materials) of metal stents on the ablation effect

**DOI:** 10.3389/fcvm.2025.1603410

**Published:** 2025-05-20

**Authors:** Zhen Wang, Ming Liang, Jingyang Sun, Jie Zhang, Yunhao Li, Fengqi Xuan, Yaling Han

**Affiliations:** ^1^ College of Medicine and Biological Information Engineering, Northeastern University, Shenyang, China; ^2^ Department of Cardiology, General Hospital of Northern Theater Command, Shenyang, China; ^3^ National Key Laboratory of Frigid Zone Cardiovascular Diseases, Shenyang, China; ^4^ Department of Cardiology, Tianjin Chest Hospital, Tianjin, China

**Keywords:** arrhythmia, pulsed electric field ablation, computer simulation, region of ablation, coronary artery, metallic stents

## Abstract

**Background:**

Arrhythmias are a common group of cardiovascular diseases, and in recent years, pulsed electric field ablation technology has made great progress in the treatment of arrhythmias. However, the effect of different materials and shapes of cardiac stents on the ablation effect at the target site during epicardial ablation has not been effectively studied.

**Methods:**

The ablation targeting position was modeled and simplified, the ablation catheter and cardiac stent were restored in real size, and the ablation region was modeled in three dimensions using a computer. Multi-model cardiac stents were established and different materials were added to different stents, and the ablation region was effectively evaluated with a field strength of 1,000 V/cm as the contour.

**Results:**

The ablative electric field was present only at the ablation targeting site. In the computational simulation, the effective ablation width caused by the simplified metal stent was not significantly different from that of the real stent (the maximum difference in ablation width was 0.34 mm). The variation of the effective ablation area caused by different metals was small (for example, the ablation catheter was 0.5 mm away from the metal stent, and the width of the effective ablation area ranged from 9.79 mm to 10.13 mm). The maximum temperature change at the targeting site caused by the simplified stent of the scaffold was small (the maximum temperature after ablation was 43.88°C, and the maximum temperature after ablation of the real scaffold was 45.19°C).

**Conclusion:**

The simulation results show that the width of the targeted ablation area does not change greatly due to the shape and material of the metal stent, and the simplified metal stent model can effectively predict the effective ablation area. The metal stent has a certain warming effect on the ablation area, and the warming phenomenon of the real stent is more intense than that of the simplified stent, but this temperature change does not cause thermal damage to the targeting site.

## 1 Introduction

Arrhythmia is the irregular beating of the heart caused by abnormalities in the electrical conduction system of the heart, and more serious arrhythmias can cause symptoms such as palpitations, chest tightness, and even the risk of death in severe cases (1–3). In recent years, the use of pulsed electric field ablation (PFA) technology for the treatment of arrhythmia has gained widespread interest among clinical experts, and this technology utilizes the differences in electric field thresholds of different tissues in the ablation area to specifically ablate the target tissues and then block the abnormal conduction pathways of the cardiac electrical signals to treat arrhythmia (4–6). Researchers have found that the epicardial ganglion plexus plays an important role in the transmission of cardiac electrical signals, which makes it possible to treat arrhythmia with ablation of the epicardial ganglia. Computational simulations have shown that the high electrical conductivity of the ganglion plexus distorts the electric field distribution in the ablation target area (7, 8). Researchers believe that the metal stent (MS) has a higher electrical conductivity compared to the tissue in the ablation area, so the presence of MS will exacerbate the phenomenon of electric field distortion. Hogens et al. experimentally demonstrated that the presence of MS will play a certain distortion effect on the normal distribution of the ablation electric field. At the same time, subsequent researchers have found that the presence of MS also has a positive effect on the temperature increase in the ablation region (9, 10).

Recently, some researchers used computers to perform computational simulations of the phenomenon of electric field twisting and distortion and thermal distribution phenomena in the ablation region of the metal scaffold, in the computational model, the presence of MS has a certain positive effect on the temperature increase in the ablation targeting region, but this change in temperature does not produce thermal damage to the ablated tissue. The researchers used the computer to establish a more complex three-dimensional computational model, and found that in the ablation-targeted region, the width and depth of the effective ablation area compared to the simplified computational simulation results data close to the simplified computational simulation, which in turn proved the effectiveness of the simplified computational simulation (11–13). In view of the fact that it has been demonstrated that the presence of MS has a large effect on the electric field distribution and thermal distribution in the ablation region. However, previous studies have only investigated simplified metallic supports from a single material, which has left the effect of material and shape on the electric field distribution and thermal distribution yet to be effectively evaluated. Therefore, we utilize computers to simulate the MS with multi-shape and multi-material analogs. The effects of different materials and different shapes of MS on the electric field distribution and thermal distribution of pulsed electric field ablation are mainly evaluated in a three-dimensional computational model. The various effects of different MS on the ablation region were analyzed, which in turn can play a greater role in facilitating the treatment of arrhythmia by epicardial ablation with PFA.

## 2 Methods

### 2.1 Computational modeling

**Figure 1:**
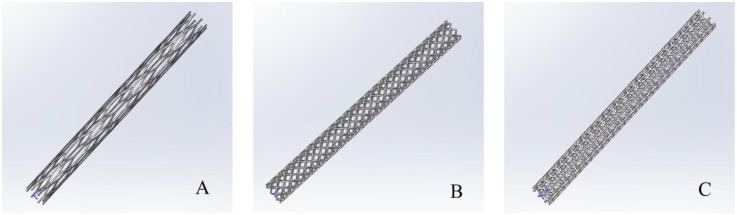
Three-dimensional model of the metal stent. In the **(A–C)** represent three different structures of metal stents.

In order to facilitate the comparison of the effect of different metal bracket models on PFA, we need to utilize Solidworks software to build a 3D model of the MS, and after the completion of the model establishment, the model is imported into Comsol for simulation and different material properties are assigned to the MS, and different MS models are shown in Figure 1. The construction of these stent models follows previously reported approaches (14, 15). It is worth noting that it has been proved in previous studies that it is effective to simulate the ablation region with reasonable simplification. Therefore, in this analog simulation study, we carried out a reasonable simplification treatment for the ablation targeting region. In this computational simulation, the tissues in the ablation region were stratified, and according to the real structure of each tissue in the human body, the ablation model was simplified into the saline layer, the fat layer, the myocardial layer, and the blood layer (the thickness of the saline layer was 0.5 mm, the thickness of the fat layer was 4.3 mm, the thickness of the myocardial layer was 2.7 mm, and the thickness of the blood layer was 40 mm) (11, 13, 16–18).

**Figure 2:**
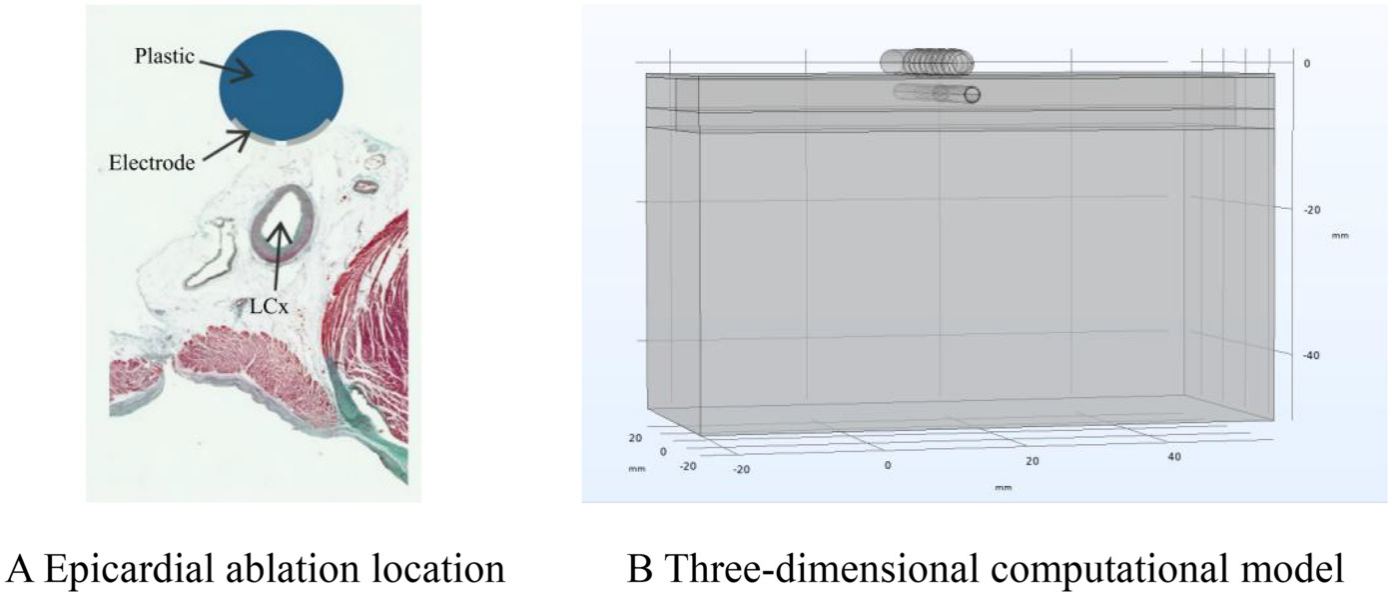
Ablation model. Panel **(A)** shows a schematic diagram of the ablation location. Adapted with permission from “Physical situation modelled in the study” by Ana González-Suárez, Juan J Pérez, Barry O'Brien and Adnan Elahi, licensed under CC BY 4.0. Panel **(B)** shows a schematic diagram of the finite element calculation model.

In the calculation model, to simulate the calculation results more in line with the actual real vascular state of the human body, we set the lipid layer inside the coronary artery to simulate the presence of plaque in the vessel. At the same time, the ablation catheter was placed on the upper side of the lipid layer according to the positional status of the catheter in the true ablation state of the epicardium. The top side of the model is the saline layer, and the ablation catheter (diameter 3.98 mm, electrode length 3.18, electrode spacing 7.16 mm.) is placed in the saline layer, and the ablation electrodes are near the fat layer, where the saline layer acts as a virtual electrode, which ensures that the ablation energy can be effectively transmitted to the target area of ablation (13, 19–21). The ablation model is shown in Figure 2.

### 2.2 Pulse parameters and boundary conditions

**Figure 3:**
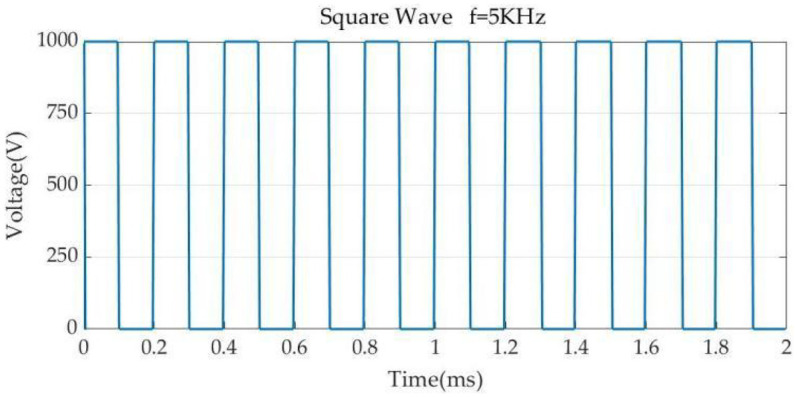
Pulse parameters. Pulse width 100 μs, pulse interval 100 μs.

In the 3D model, we separate the boundary conditions, and for the model electrical boundary conditions, set the ablation catheter release voltage to 1,000 V, and the pulse time interval is set to 100 μs, and the time of each pulse is 100 μs. meanwhile, set the ablation electrode spacing insulator to 0 V, and set the model bottom surface to 0 V (dispersive electrode.), and simulate the current flow in the flow in the model. In order for the ablation energy to be effectively transferred in the model, the model surface current was set to 0 A (13, 22, 23). The pulse parameters, adopted from previously reported studies (11, 22), are shown in Figure 3.

In the model, we evaluate the temperature of the ablation region. To accurately evaluate the effect of different shapes and materials of the metal support on the temperature of the ablation region, the temperature boundary conditions are set in the computational model. The thermal convection coefficient of 20 W/m^2^K is set at the air-electrode interface (the ambient temperature is 21°C). A thermal convection coefficient of 1,417 W/m^2^K was applied at the myocardium-blood interface (blood temperature was 37°C). Simulating the circulatory state in blood flow (24.4 cm/s). In the condition where there is no stent inside the coronary artery, we simplify the simulated state by simplifying it with a constant blood flow rate of 50 cm/s, setting the thermal convection coefficient in the calculation to 63.19 W/m^2^K (16, 23–25). At the same time, we added the probe function to the computational model to facilitate the recording of the trend of temperature increase with pulse voltage change during the computational simulation.

### 2.3 Material properties

The content of this study belongs to the electro-thermal coupling problem, in which we chose COMSOL software for numerical simulation and analyzed the results by using the post-processing function of the software. The conductivity of biological tissues plays an important role in the accuracy of the computer simulation results, researchers found that the conductivity of biological tissues shows an S-shaped distribution with the issuance of the electrical pulse energy, which is because after the biological tissues have been electrically stimulated, the permeability of the cells is changed, which leads to a gradual increase in the conductivity of the tissues, and in the present study, we use the Sigmoid function to simulate this change in the conductivity (26, 27). To calculate the accuracy of the results, the change in tissue temperature in the study has a large effect on the conductivity, so we set the conductivity to increase by 2% with the change of temperature to consider the effect of temperature. The various electrical and thermal physical parameters to be used in the simulation are shown in Table 1 (11, 28–32).

$$\sigma (E,T)=(\sigma_{0}+\frac{\sigma_{1}-\sigma_{0}}{1+10e^{-}\frac{\left(|E|-58,000\right)}{3000}})\cdot 1.02^{T-37}$$

**Table 1:** Calculated parameters of the model.

Mlement	$\sigma_{0}$ (S/m)	$\sigma_{1}$ (S/m)	K (W/m k)	C (J/Kg·k)	ρ (Kg/m^3^)
No. 1 metal	7.4e^6^		15	480	8,000
No. 2 metal	4.032e^6^		44.5	475	7,850
Electrode	4.6e^6^		71	132	21,500
Poyurethane	1e^−5^		23	1,050	1,440
Saline	1.392		0.628	4,148	980
Lipid plaque	0.09		0.3	2,400	950
Vessel	0.4	0.67	0.5	3,400	1,100
Myocardium	0.0537	0.281	0.56	3,686	1,081
Blood	0.7	0.748	0.52	3,617	1,050
Fat	0.0377	0.0438	0.21	2,348	911

In the formula, with σ_0_ and σ_1_ table is the different conductivity of biological tissues, this is mainly because in biological tissues, the size of the conductivity is related to the size of the pores of the cell membrane. Before the pulse energy is applied to the biological tissue, the pores above the biofilm have not been formed yet, at this time, the current flows only on the surface of the biological tissue, and the cell membrane is like an insulating membrane at this time, so the conductivity of the biological tissue is low before the pores of the biofilm are not formed, and in this study, we use σ_0_ to indicate the conductivity of the biological tissue in the current state. The study proved that when the biological tissue is subjected to high-frequency pulse energy. The cell membrane of biological tissues will form pores at this time, and these pores will allow the current to pass through the cytoplasm, thus leading to a change in the electrical conductivity of biological tissues, at this time, σ_1_ is used to indicate the electrical conductivity of biological tissues in the current state (13, 33, 34). For non-biological tissues (electrodes, saline, metal scaffolds.), in the present study, we do not consider the change in conductivity that occurs before and after the application of pulse energy.

### 2.4 Controlling equations

In the present study, we utilized COMSOL software for 3D modeling of biological tissues as well as catheters, a research method that has been proven to be reliable in previous studies. We used the self-contained function of COMSOL software to mesh the 3D model, and it is worth noting that, in order to perform more detailed modeling of the metal stent, we used Solidworks software to model the MS, and then imported the model into COMSOL software for unified meshing and computational simulation (13, 26, 35). For the current module, in our study, we introduced Laplace's system of equations to simulate the model, which is suitable for solving the control equations when the model has no internal power supply, as shown above.

$$\left\{ \begin{array}{c} \nabla \cdot (\sigma \nabla V)=0\\ E=-\nabla V\\ J=\sigma E \end{array}\right.$$

In the formula, it denotes the conductivity (S/m); E denotes the electric field strength (V/m); V denotes the pulse voltage (V); J is the current density (A/m2); and the specific values used in the calculation are given in Table 1. For the bioheat problem generated when biological tissues are subjected to pulsed energy, we add the bioheat equation module to the simulation for calculation, and the application of the probe function in the model will be helpful for us to study the thermal side effects induced by PFA energy (11, 36).

$$\left\{ \begin{array}{c} \rho c_{p}\frac{dT}{dt}=\nabla \cdot (k\nabla T)+Q+Q_{b}+Q_{met}\;\\ Q_{b}=p_{b}c_{b}q_{b}\cdot (T_{b}-T_{t})\\ Q=\sigma |E{|}^{2} \end{array}\right.$$

In the formula, denotes density (Kg/m^3^); cp denotes tissue-specific heat (J/Kg·K); T denotes temperature (℃); t denotes time (s); k denotes tissue thermal conductivity (W/m·K); Q denotes heat source generated by the pulse energy, which is related to the electric field strength and conductivity (W/m^3^); Qb denotes heat loss caused by blood flow (W/m^3^); pb denotes blood density (Kg/m^3^); cb denotes specific heat of blood (J/Kg·K); qb denotes blood flow (m/s); Tb denotes blood temperature (℃); Tt denotes tissue temperature (℃); and Qmet denotes heat loss due to biological metabolism (W/m^3^). The numerical quantities used in the equations are given in the text and Table 1.

### 2.5 Analysis of results

In this study, we performed simulation calculations for the PFA region using COMSOL finite element software to evaluate the electric field distribution during pulsed electric field ablation, and we also used the bio-thermal module to evaluate the temperature changes in the ablation targeting region. In this study, we performed simulation calculations for different materials and shapes of MS in various scenarios: (1) Setting up different distances between the ablation catheter and the metal stent. (2) Importing different shapes of M for different computational models. (3) Setting up different metal materials for MS in computational models. (4) In the simulation calculation, we did not change the parameters of biological tissues such as myocardium and blood, which is to facilitate the comparison of the electric and thermal effects of different MS on the ablation area. In the post-processing stage of the results, we utilized the electric field strength of 1,000 V/cm as the threshold contour of the ablation region to assess the effective range of the ablation targeting region (11, 37). This is because it has been reported in previous studies that electric field strengths greater than 1,000 V/cm under these pulsed conditions will result in irreversible damage to the myocardium. We did not make any changes to the width and spacing of the pulse voltages in our study because the bioheat generated by the pulse energy was found to be related only to the pulse width and number of pulses in previous studies.

## 3 Results

**Figure 4:**
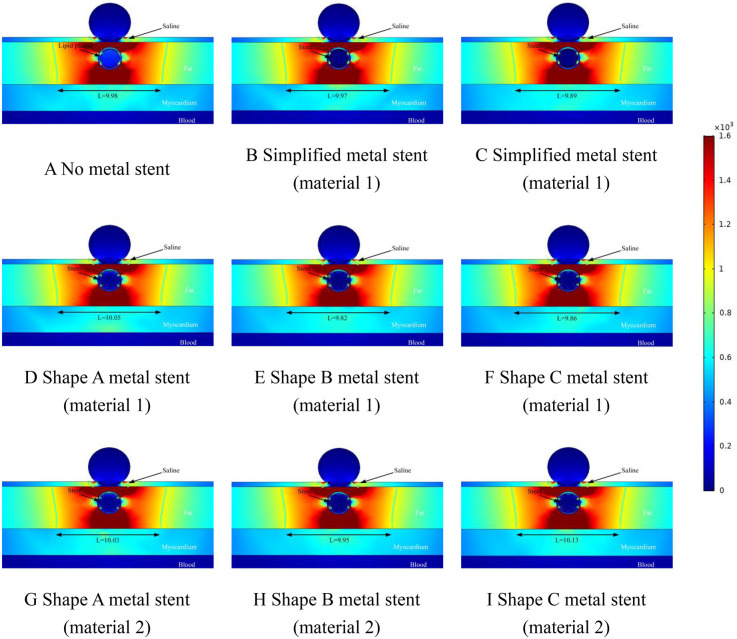
Effect of pulsed electric field ablation electric field distribution when the spacing between the ablation catheter and the metal stent is 0.5 mm. Panels **(A–I)** show the effect of different shapes (materials) of metal supports on the electric field distribution. The metal stent shape A, shape B and shape C are shown in Figure 1, and the material properties are shown in Table 1.

We performed ablation simulations with different shapes of MS inside the coronary arteries, which also included the absence of MS inside the coronary arteries, which may occur during clinical ablation of patients for arrhythmia treatment with blockage inside the coronary arteries but without a metallic stent inside the coronary arteries. Therefore, in this case, we only set up the lipid layer in the model. Meanwhile, the focus of this simulation is on the effect of MS on the ablation effect, so we simulate different MSs, and at the same time, different MSs are assigned to different MSs. In the field intensity distribution and temperature distribution diagrams, Figure 4A shows that there is no MS inside the coronary artery, and Figures 4B,C show that the coronary artery has the MS of the simplified model, and the difference lies in the different materials assigned to the metal stent. Different Figures 4D–F show the ablation effect of ablating three different shapes of MS, and Figures 4G–I show the three different shapes of MS endowed with a second metallic material (the three shapes of MS are shown in Figure 1, and the parameters of the metallic material properties are shown in Table 1).

### 3.1 Electric field distribution

The results of this simulation are demonstrated in Figure 4, where the distance between the ablation catheter and the coronary artery is 0.5 mm. It can be obtained from the Figure that the presence of the coronary artery distorts the ablation electric field (the presence or absence of the MS has no effect on this phenomenon.). The presence of a metallic stent in the coronary artery causes the ablation field to be distorted (the presence or absence of a metallic stent does not affect this phenomenon). MS inside the coronary arteries did not have a large effect on the effective ablation of the pulsed electric field, and in Figure 4, the maximum difference in the effective ablation width between the absence of MS and the presence of MS was 0.15 mm (the ablation width was 9.98 mm in Figure 4A and 10.13 mm in Figure 4I).

This suggests that the presence of intracoronary MS does not have a large effect on the ablation width of the pulsed electric field. The large difference in effective ablation width between simplified MS and real MS was 0.24 mm (ablation width of 9.89 mm in Figure 4C and 10.13 mm in Figure 4I.). This suggests that different shapes of MS do not have a large effect on the effective ablation width during pulsed-field ablation, and it also suggests that we can reliably calculate the effective ablation width by simplifying the model of MS when performing simulations. When the coronary arteries have MS, the electric field strength inside the coronary arteries is smaller than that without MS, which is not related to the shape and material of the MS (without MS, the field strength inside the coronary arteries is 280 V/cm; with MS, the field strength inside the coronary arteries is 23 V/cm).

**Figure 5:**
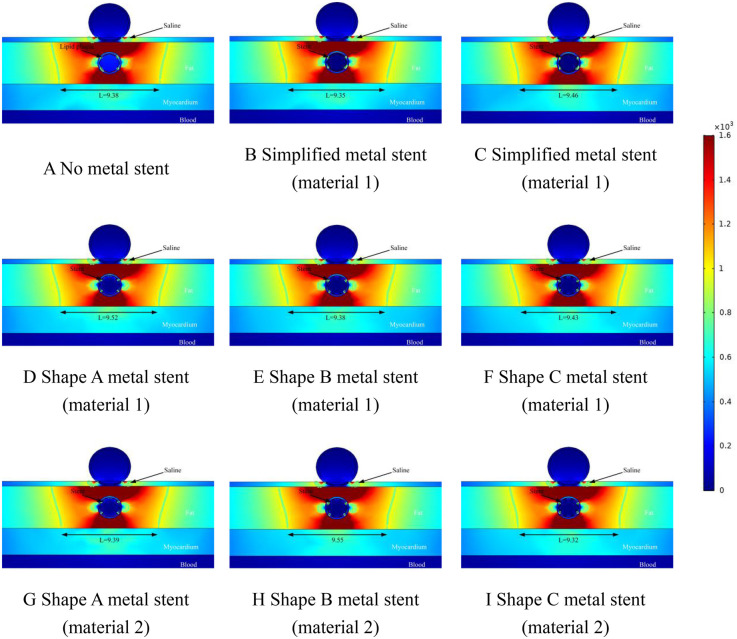
Effect of pulsed electric field ablation electric field distribution when the spacing between the ablation catheter and the metal stent is 1.0 mm. Panels **(A–I)** show the effect of different shapes (materials) of metal supports on the electric field distribution. The metal stent shape A, shape B and shape C are shown in Figure 1, and the material properties are shown in Table 1.

Figure 5 shows a simulation of the electric field distribution at a distance of 1 mm between the ablation catheter and the coronary artery. In Figure 5, the cold spots of the electric field appeared at the same locations as in Figure 4, which indicates that the location of the cold spots did not correlate with the location of the coronary artery in the fat layer. Comparison of Figure 4 gives the effective ablation width of the ablated regions in Figure 5 (regions greater than 1,000/cm) is smaller than the ablation width shown in Figure 4. It is worth noting that the effective ablation widths of the ablation regions that we have identified in the Figure are the smallest values measured (near the myocardial layer.). In Figure 5, the maximum difference in effective ablation width between the absence and presence of MS is 0.17 mm (9.38 mm in Figure 5A and 9.55 mm in Figure 5H). The presence of MS does not have a large effect on the ablation width, which is the same as the ablation results shown in Figure 4. The maximum difference in the ablation area caused by the same shape of MS and different materials was 0.17 mm (ablation width of 9.38 mm in Figure 5E and 9.55 mm in Figure 5H). In this epicardial pulsed-field ablation process, the material of the intracoronary MS had less influence on the effective ablation area width.

**Figure 6:**
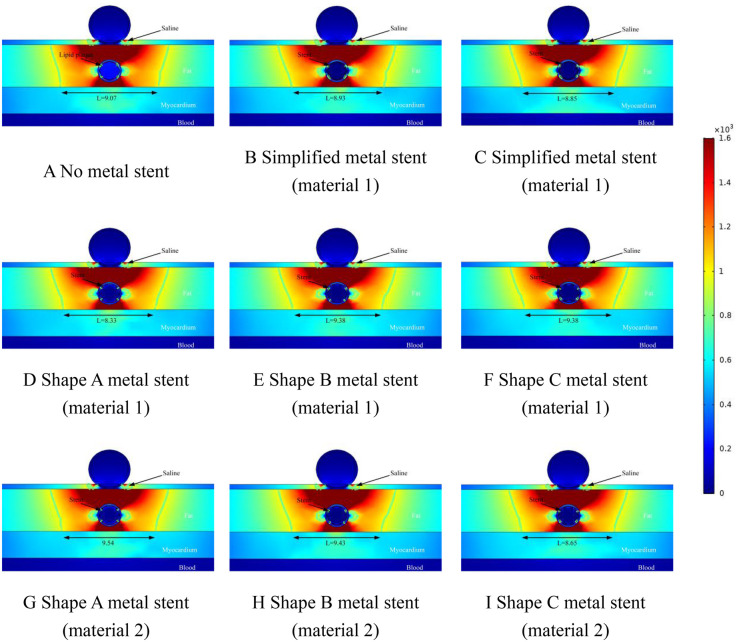
Effect of pulsed electric field ablation electric field distribution when the spacing between the ablation catheter and the metal stent is 1.5 mm. Panels **(A–I)** show the effect of different shapes (materials) of metal supports on the electric field distribution. The metal stent shape A, shape B and shape C are shown in Figure 1, and the material properties are shown in Table 1.

The ablation effect at a distance of 1.5 mm between the ablation catheter and the coronary artery is shown schematically in Figure 6. From Figure 6, it can be obtained that the position of the cold spot of the electric field in the ablation region shifted further downward with the position of the coronary artery and the position of the cold spot was located on the left and right sides of the coronary artery, and the position of the cold spot was similar to that in which the cold spot appeared in Figures 4, 5. The width of the effective ablation area was further reduced with the distance between the ablation catheter and the coronary artery (e.g., the width of the ablation area was 9.38 in Figure 5A and 9.07 in Figure 6A). In Figure 6, the maximum difference between the effective ablation width without MS and with MS was 0.47 mm (9.38 mm ablation width in Figure 6A and 9.54 mm ablation width in Figure 6G.). The maximum difference in effective ablation width caused by MS with different materials of the same shape was 1.21 mm (9.38 mm ablation width in Figure 6D, 9.54 mm ablation width in Figure 6G.). Similar to Figures 4, 5, the value of the electric field inside the coronary arteries was much less than 1,000/cm, which suggests that this indicates that the pulsed electric field does not cause damage to the coronary arteries under this pulse condition.

### 3.2 Temperature distribution

**Figure 7:**
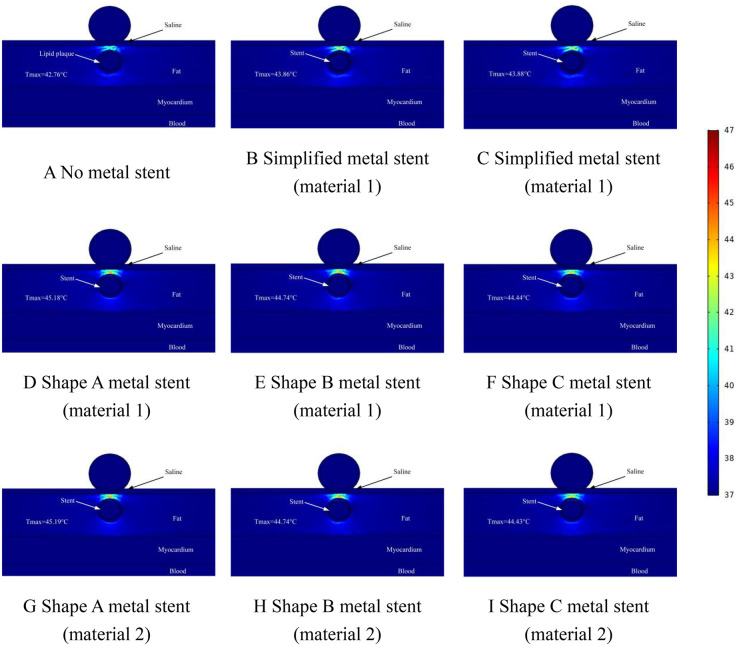
Effect of temperature distribution of pulsed electric field ablation when the spacing between ablation catheter and metal stent is 0.5 mm. Panels **(A–I)** show the effect of different shapes (materials) of metal supports on the temperature distribution. The metal stent shape A, shape B and shape C are shown in Figure 1, and the material properties are shown in Table 1.

**Figure 8:**
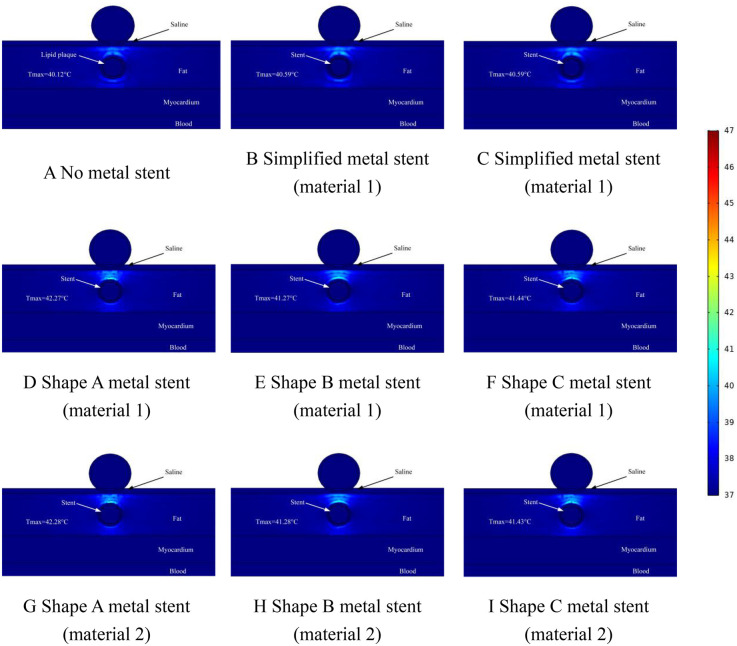
Effect of temperature distribution of pulsed electric field ablation when the spacing between ablation catheter and metal stent is 1.0 mm. Panels **(A–I)** show the effect of different shapes (materials) of metal supports on the temperature distribution. The metal stent shape A, shape B and shape C are shown in Figure 1, and the material properties are shown in Table 1.

**Figure 9:**
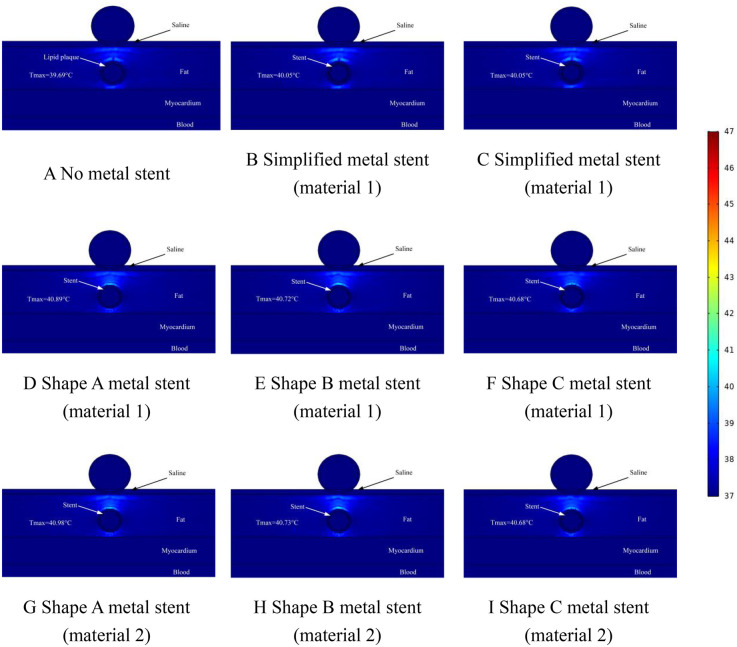
Effect of temperature distribution of pulsed electric field ablation when the spacing between ablation catheter and metal stent is 1.5 mm. Panels **(A–I)** show the effect of different shapes (materials) of metal supports on the temperature distribution. The metal stent shape A, shape B and shape C are shown in Figure 1, and the material properties are shown in Table 1.

Too high a temperature in the ablation region will cause thermal damage in the ablation region, so the thermal distribution caused by MS inside the coronary artery is another focus of this study. In the temperature study, we set the distance between the ablation catheter and the coronary artery to be 0.5 mm, 1 mm, and 1.5 mm in three states, which are represented in Figures 7–9, respectively, and it is worth noting that, in the graded distribution study, the cross-section we took was the same as that of the electric field distribution above, which was to study both the electric field distribution and the temperature distribution condition at the same time in the same cross-section. In the measurement of the temperature distribution in the ablation region, we utilize the probe function that comes with the software to measure the maximum temperature of the cross-section in the ablation region.

The temperature distribution in the ablation region when the distance between the ablation catheter and the coronary artery was 0.5 mm is shown schematically in Figure 7. From Figure 7, we can see that the maximum temperature in the ablation region was 42.76°C when there was no MS inside the coronary artery (as shown in Figure 7A). When MS was present inside the coronary artery, the maximum temperature of the ablation region was 45.19°C (as shown in Figure 7G). The maximum difference in temperature change in the ablation region caused by MS of the same shape and different metallic materials was 0.01°C, and the maximum difference in temperature change in the ablation region caused by MS of different shapes and the same metallic materials was 0.76°C (as shown in Figures 7G,I). It is worth noting that the presence or absence of MS inside the coronary arteries affected the temperature effect by a maximum difference of 2.43°C. When the MS in the model was a simplified shape MS, the maximum temperature in the ablation region was 43.88°C (as shown in Figure 7C). When the distance between the ablation catheter and the coronary artery was 0.5 mm, the maximum temperature difference between the ablation region caused by the simplified MS and the real MS was 1.33°C (as shown in Figures 7B,G).

In this simulation study, we adjusted the distance between the ablation catheter and the coronary artery. The temperature distribution of the ablation region when the ablation catheter was 1 mm away from the coronary artery is shown in Figure 8. From the graph, it can be learned that when there was no MS inside the coronary artery, the highest temperature in the ablation region was 40.12°C (as shown in Figure 8A), when there was MS inside the coronary artery, the highest temperature in the ablation region was 42.28°C (as shown in Figure 8G). It is worth noting that the location of the highest temperature in the ablation region appeared in the same area as the area where the hot spot of the electric field (electric field strength greater than 1,600 V/cm.) appeared, which indicates that in the ablation region, there is a relationship between the level of the electric field strength and the location of the temperature distribution. In the simplified MS simulation calculation, the highest temperature in the ablation region is 40.59°C (shown in Figure 8C). The maximum temperature in the ablation region of the simplified MS is lower than the temperature enhancement effect induced by the real MS in the ablation region, with a maximum difference of 1.69°C, which is similar to the results of the distribution of the ablation region in Figure 7 above (as shown in Figures 8B,G).

Figure 9 shows the temperature distribution in the ablation area when the distance between the ablation catheter and the coronary artery was 1.5 mm. The Figure shows that the highest temperature in the ablation region was 39.69°C when there was no MS inside the coronary artery, and the location of the highest temperature appeared to be on the upper side of the coronary artery, which was consistent with the temperatures that appeared in Figures 7, 8 above (as shown in Figure 9A). The highest temperature in the ablation region of the pulsed electric field was less than that shown in Figures 7, 8, which indicates that the temperature in the ablation region caused by the MS inside the coronary artery changed with the change in the distance between the ablation catheter and the coronary artery. When MS was present inside the coronary artery, the maximum temperature in the ablation region was 40.98°C (as shown in Figure 9G), which was a temperature increase of 1.29°C compared with the temperature in the absence of MS in the coronary artery (as shown in Figures 8A,G). In addition, we found that the maximum temperature induced by simplified MS in the ablation region was 40.05°C, and the temperature increase induced by simplified MS was not as dramatic as that induced by MS stenting, a result similar to that shown in Figures 7, 8 above.

## 4 Discussion

### 4.1 Main findings

In this study, we made a detailed study of the electric field distribution and thermal distribution caused by PFA ablation in the epicardium, and we designed different shapes of MS in the 3D computational model (as shown in Figure 1). We made a multi-distance simulation of the distance between the ablation catheter and the coronary artery, which is mainly considering the differences in the thickness of the epicardial fat layer in different patients, in addition, it was found that patients with coronary artery disease are greater than the thickness of the epicardial fat layer in the healthy population (38, 39). Meanwhile, the simplified MS and MS-free coronary arteries were set up in different computational models to make a comparative study, to consider the changes in electric field distribution and temperature distribution caused by different metal materials to the ablation area, different metal materials were assigned to the MS for the comparison of the ablation results in this study.PFA is the use of the inconsistency in the sensitivity of different tissues of the organisms to the strength of the electric field for the specific tissue ablation, and biological tissues undergo damage as a result of the action of electric field strength (33, 40, 41).

Recently, however, it has been shown that the presence of MS in the organism will have a positive effect on the temperature elevation in the ablation region, and it has also been found that the presence of MS in the organism will have a distorting effect on the normal electric field distribution of the PFA (10, 11, 42). However, so far no researcher has analyzed the shape and material of MS in the organism in a comparative aspect. According to the information we reviewed, our study is the first one to investigate the effect of MS in epicardial PFA on the electric field distribution and the thermal distribution in the ablation targeting region, starting from the shape and material of MS in the intracoronary arteries. In this study, we performed a three-dimensional computational simulation of the region of interest of the ablation catheter using three-dimensional modeling software and finite element analysis software, and we also performed a multi-distance setup for the distance between the ablation catheter and the coronary artery. The main findings of this calculation in this study are shown below.

1. At the same ablation position, the maximum difference in the width of the ablation area caused by different shapes of MS with the same material is 0.34 mm. When the MS shape is the same, the maximum difference in the width of the ablation area caused by different materials is 0.27 mm. The shape and material of the MS have limited changes in the size of the ablation area effectively ablated by the pulsed electric field.
2. When MS is present inside the coronary artery, the value of the internal electric field of the coronary artery is only twenty or so, and when there is no MS inside the coronary artery, the value of the internal electric field of the coronary artery is two hundred or so, and this conclusion has nothing to do with the shape and material of the MS.
3. The simplified shape of the MS can effectively predict the effective ablation region size for PFA ablation, which suggests that the MS can be effectively simplified in the simulation calculation when the ablation region size is predicted.
4. The presence of intracoronary MS can have a positive effect on the temperature elevation in the ablation region, which can be demonstrated by both simplified MS and different shapes of real MS, but the phenomenon of temperature elevation induced by real MS is more pronounced in comparison to simplified stents.
5. In the PFA-targeted ablation region, the two regions with the highest values of electric field distribution and temperature distribution overlap in the region between the ablation catheter and the coronary artery. At the same time, the locations of the lowest values of both are located in the region on both sides of the coronary arteries.
6. When the distance between the ablation catheter and the coronary artery was constant, the maximum difference in the temperature rise induced by different shapes of MS and MS of the same material in the ablation region was only 0.76°C. The difference in temperature rise induced by different shapes of MS and MS of the same material in the ablation region was only 0.76°C. The shape of MS in the study caused extremely limited thermal changes in the ablation region.

### 4.2 Electric field distribution analysis

When a coronary artery is present in the PFA-targeted ablation area, the distribution of the electric field in the ablation area is somewhat distorted, and this phenomenon is not related to the presence or absence of MS within the coronary artery. In addition, the distance between the ablation catheter and the coronary artery does not affect the distortion of the electric field in the ablation region. In our simulation study, the effective ablation area of the pulsed electric field was limited to the epicardial fat layer only and did not damage the myocardial layer, and if the pulse energy is needed to damage part of the myocardium, it is necessary to change the pulse width and amplitude of the pulsed electric field (41, 43). The value of the electric field inside the coronary arteries is much smaller than that in the targeted ablation area, which is not related to the presence or absence of MS inside the coronary arteries.

In the present study, different simulations of the shape and material of the MS were analyzed and it was found that the width of the PFA-targeted ablation area did not correlate with the presence or absence of MS inside the coronary arteries. In the study, for the same shape of MS, there was a large difference in the conductivity of different materials, but this did not have a large impact on the ablation area, which may be because: (1) MS itself occupies a relatively small cross-sectional area in the ablation area; and (2) MS spaced between the internal coronary arteries and the ablation catheter in the presence of lipid plaques, coronary arteries, adipose, and other tissues, and the material properties of these tissues were not altered. These factors resulted in limited changes in the area of the targeted ablation area affected by changes in MS material.

### 4.3 Temperature distribution analysis

With the multivariate setting of the distance between the ablation catheter and the coronary artery in our computational model, the computational simulation found that the MS inside the coronary artery had a positive effect on the temperature elevation in the ablation region, a finding that was not related to what kind of changes in the shape and material of the MS inside the coronary artery occurred. It is worth noting that the temperature elevation caused by real MS was more pronounced in the computational simulations, probably due to the discontinuity of the mesh junction of real MS, which forms eddy current losses inside the electric field after penetration and therefore leads to more heat generation (44, 45). However, regardless of the changes in the shape and material of the MS inside the coronary arteries, the maximum temperature induced by the MS in the ablation region was 45.19°C, and such a temperature change was much smaller than the thermal damage value of 55°C for biological tissues. Therefore, the pulsed electric field does not cause thermal damage to the biological tissues in the targeted ablation region (11, 46, 47).

In our computational simulation, the change in the distance between the ablation catheter and the coronary artery had a large effect on the elevated temperature of the ablation region. When the distance between the ablation catheter and the coronary artery was not 0.5 mm, the average temperature of the ablation region induced by the real MS was 44.79°C. When the distance between the ablation catheter and the coronary artery was not 1.0 mm, the average temperature of the ablation region induced by the real MS was 41.86°C. When the distance between the ablation catheter and the coronary artery was not 1.5 mm, the mean temperature of the ablation region induced by real MS was 40.78°C. When the distance between the ablation catheter and the coronary artery was varied, the maximum temperature change in the ablation region was 1.30°C and 2.91°C, and the average temperature change in the ablation region was 1.08°C and 2.93°C. However, it is worth noting that when the distance between the ablation catheter and the coronary artery was 0.5 mm, the ablation catheter and the coronary artery were in infinite proximity, which also indicates that the temperature generated in the ablation region is in the highest environment when using PFA for epicardial ablation at this parameter, which also proves that the PFA technique does not produce thermal damage to the ablation region.

In this study, we investigated epicardial fat thickness by referencing previous literature and established a computational model for the thickness of each tissue layer. Notably, we did not alter the fat thickness in this study, as our primary focus was the ablative effect of the stent on the surrounding ablation region. However, we adjusted the distance between the ablation catheter and the stent using computer simulation, indirectly simulating changes in fat layer thickness. The thicknesses of the myocardial and blood layers were also based on values reported in previous studies (48, 49). This computational simulation is an *a priori* study, and the selected parameters possess a certain degree of generalizability. Therefore, our findings may have potential clinical relevance.

We consulted previous studies in an attempt to match stent material with stent geometry. However, this attempt did not yield definitive conclusions, potentially due to methodological limitations or other confounding factors. In this study, the material parameters assigned to different stents were based on values reported in previous literature (14, 15, 50). Due to certain limitations, we were unable to perform experimental validation of the computational results. Nevertheless, we reviewed relevant literature, including studies on the effects of stents on electric field distribution and animal experiments validating ablation outcomes, which to some extent support our findings (10, 47, 51).

In this study, a threshold of 1,000 V/cm was selected to represent tissue damage. This threshold may differ from values reported in other studies, as tissue damage is influenced by various parameters such as the number of pulses, pulse width, and pulse interval. Therefore, we adopted the 1,000 V/cm threshold to align with the pulse parameters used in our ablation model (52, 53). To ensure the rigor of the simulation, we conducted mesh sensitivity tests for tissue discretization. Excessively fine meshes led to a substantial increase in computational cost. Conversely, overly coarse meshes generated low-quality elements that negatively affected model convergence. However, mesh density had minimal impact on the overall ablation outcomes. Thus, the ablation results obtained from this computational model can be considered reasonably reliable.

In our study, the presence of the stent resulted in a cold spot within the ablation zone, where the electric field strength failed to reach the 1,000 V/cm threshold. This insufficient field strength can lead to incomplete ablation, potentially resulting in arrhythmia recurrence. However, our previous findings indicated that adjusting the angle between the ablation catheter and the stent alters the location of the cold spot. Therefore, optimizing the catheter–stent orientation may improve ablation efficacy in clinical practice (22). In addition, the sinus node or phrenic nerve and tissues of note during ablation, such as during ablation of the right upper pulmonary vein, should be avoided by applying high-intensity pulses and monitored intraoperatively with electrical stimulation. Finally, numerous clinical studies have confirmed the tissue selectivity of PFA. When applied appropriately, PFA enables rapid and efficient treatment of arrhythmias, offering a promising therapeutic option for affected patients (54, 55).

### 4.4 Limitations

In the present study, we did multi-shape modeling of the shape of MS inside the coronary arteries, but in the process of assigning metallic material properties to the MS model, we did not find the relevant material properties of drug-coated stents and bioavailable scaffolds in our previous study, which implies that our assignment of the MS material may be different from that of drug-coated stents. Differences in conductivity may cause differences in the area of the targeted ablation area, but this amount of change was not significant in our study. In addition, the lack of material properties of drug-coated stents may make a difference in our assessment of the temperature in the ablation area, but the magnitude of the specific heat capacity of the material has a decisive effect on the temperature of the ablation area during the ablation process, and the stent used in our study was a bare metal stent, which implies that the amplification of the specific heat capacity of the stent in our simulation calculations makes it possible to achieve the same results in practical clinical application, the maximum value of MS-induced bioheat may be smaller than the calculated value simulated in this study. Although we performed simulations from multiple perspectives, such as the distance between the ablation catheter and the coronary artery, the shape of the MS, and the MS, this study was based on simulations from a physical point of view, and therefore, we recommend that a large number of animal experiments be performed to validate the results of our study in clinical applications.

## 5 Conclusion

It has been demonstrated that the presence of coronary MS may have some effect on the pulsed electric field targeting the ablation region, but no study has ever investigated the specific effect of MS on the ablation region from the perspective of the shape and material of MS. In this paper, for the first time, we have made a detailed study of the effects of MS of different shapes and materials on the electric field distribution and thermal distribution in the ablation region using a three-dimensional computational model. Our results show that the presence of MS inside the coronary arteries has an elevating effect on the temperature of the ablation region, which is not related to the shape and material of the MS. It was also found that real shape (true shape) MS caused a higher temperature elevation effect in the ablation region than simplified MS, but this temperature elevation did not cause thermal damage to the biological tissues in the ablation region. Finally, the width variation induced by different shapes and materials of MS inside the coronary artery in the ablation region is extremely limited, which means that the effect of MS on the width of the ablation region can be neglected during PFA ablation. It is worth noting that our results were simulated from a physical point of view, and in the future, we would welcome researchers to validate our findings by performing animal experiments on the simulation results.

## Data Availability

The raw data supporting the conclusions of this article will be made available by the authors, without undue reservation.
